# Persistent activation of microglia and NADPH drive hippocampal dysfunction in experimental multiple sclerosis

**DOI:** 10.1038/srep20926

**Published:** 2016-02-18

**Authors:** Massimiliano Di Filippo, Antonio de Iure, Carmela Giampà, Davide Chiasserini, Alessandro Tozzi, Pier Luigi Orvietani, Veronica Ghiglieri, Michela Tantucci, Valentina Durante, Ana Quiroga-Varela, Andrea Mancini, Cinzia Costa, Paola Sarchielli, Francesca Romana Fusco, Paolo Calabresi

**Affiliations:** 1 Clinica Neurologica, Dipartimento di Medicina, Università degli Studi di Perugia, Ospedale Santa Maria della Misericordia, S. Andrea delle Fratte, 06132 Perugia, Italy; 2 IRCCS, Fondazione Santa Lucia, via del Fosso di Fiorano 64, 00143, Rome, Italy; 3 Sezione di Fisiologia e Biochimica, Dipartimento di Medicina Sperimentale, Università degli Studi di Perugia, S. Andrea delle Fratte, 06132 Perugia, Italy

## Abstract

Cognitive impairment is common in multiple sclerosis (MS). Unfortunately, the synaptic and molecular mechanisms underlying MS-associated cognitive dysfunction are largely unknown. We explored the presence and the underlying mechanism of cognitive and synaptic hippocampal dysfunction during the remission phase of experimental MS. Experiments were performed in a chronic-relapsing experimental autoimmune encephalomyelitis (EAE) model of MS, after the resolution of motor deficits. Immunohistochemistry and patch-clamp recordings were performed in the CA1 hippocampal area. The hole-board was utilized as cognitive/behavioural test. In the remission phase of experimental MS, hippocampal microglial cells showed signs of activation, CA1 hippocampal synapses presented an impaired long-term potentiation (LTP) and an alteration of spatial tests became evident. The activation of hippocampal microglia mediated synaptic and cognitive/behavioural alterations during EAE. Specifically, LTP blockade was found to be caused by the reactive oxygen species (ROS)-producing enzyme nicotinamide adenine dinucleotide phosphate (NADPH) oxidase. We suggest that in the remission phase of experimental MS microglia remains activated, causing synaptic dysfunctions mediated by NADPH oxidase. Inhibition of microglial activation and NADPH oxidase may represent a promising strategy to prevent neuroplasticity impairment associated with active neuro-inflammation, with the aim to improve cognition and counteract MS disease progression.

Multiple sclerosis, one of the main causes of non-traumatic neurological disability in young adults, is a disorder of the central nervous system (CNS) characterized by both inflammatory demyelination and early pathogenic mechanisms involving neurons and synapses^1^. Accordingly, multiple sclerosis usually starts with a relapsing remitting course but, over time, most patients develop progressive neurological deficits occurring independently of acute clinical attacks^1^.

Cognitive impairment is common in multiple sclerosis, with prevalence rates ranging from 43% to 70% and it detrimentally affects many aspects of daily and social functioning, sometimes with a major impact on patients quality of life^2^. Unfortunately, the synaptic and molecular mechanisms underlying multiple sclerosis-associated cognitive impairment are still largely unknown.

It is now well accepted that the immune system and the CNS dynamically interact in both physiological and pathological conditions and that neuroinflammation and immune molecules have the potential to influence the induction of long-term synaptic plasticity, the basis for learning, cognitive and recovery processes^3^. Accordingly, it has been proposed that an alteration of synaptic plasticity driven by immune system activation might contribute to the pathogenesis of cognitive dysfunction during the course of multiple sclerosis^3^,^4^. In particular, during experimental autoimmune encephalomyelitis (EAE), abnormalities in synaptic long-term potentiation (LTP) have been described in the hippocampus^4^,^5^, a key structure for physiological cognitive functioning that seems to be particularly vulnerable during the course of multiple sclerosis^6^.

The aim of the present study was to investigate the presence, and the underlying mechanisms, of hippocampal dysfunction during the remission phase of experimental multiple sclerosis, after the resolution of quantifiable motor deficits. For this reason, we utilized an experimental model of multiple sclerosis that predictably follows a clinical course reminiscent of a relapsing–remitting disease, in which remissions occur spontaneously. In particular, in the remission phase of experimental autoimmune encephalomyelitis (EAE), we investigated hippocampal plasticity and behaviour and the possible mechanisms underlying their specific alteration.

## Results

### CA1 hippocampal microglia is persistently activated in the remission phase of experimental multiple sclerosis

**Figure 1:**
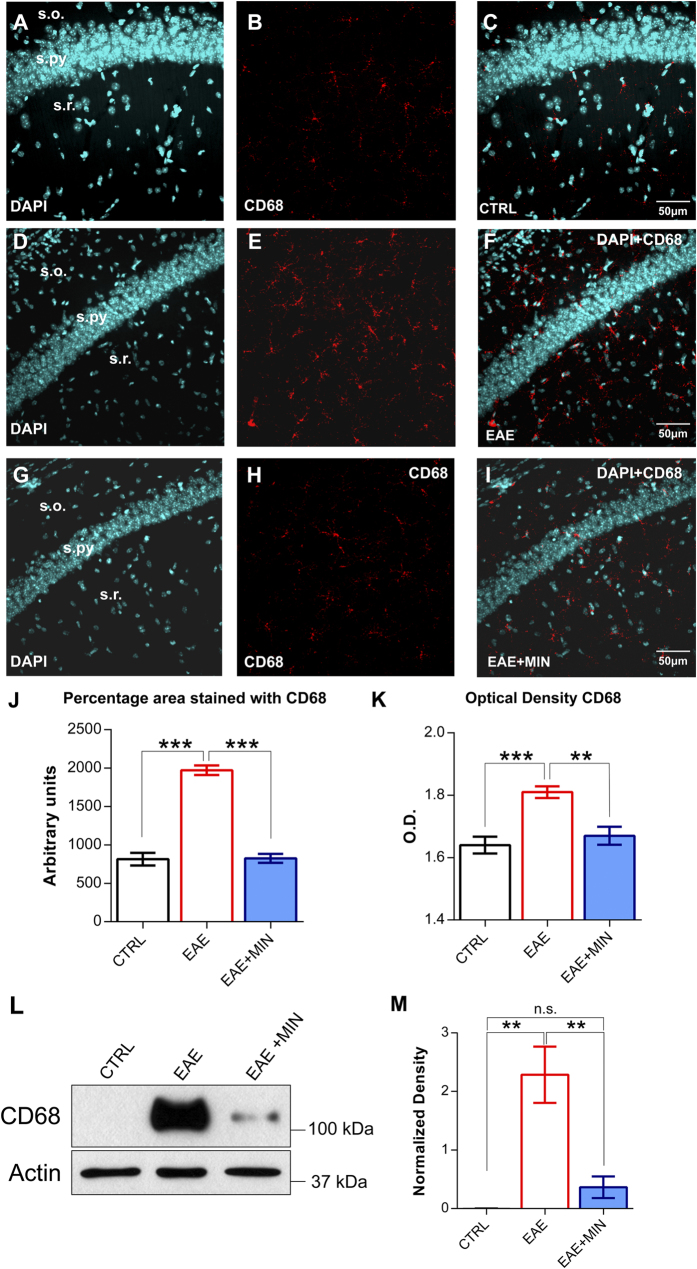
Hippocampal microglia activation in the remission phase of EAE is prevented by minocycline treatment. (**A**–**I**) Confocal laser scanning microscopy (CLSM) images of double-label immunofluorescence for DAPI (visualized in blue) and CD68 (visualized in red-cy3 fluorescence) in the CA1 hippocampal region from control mice (**A**–**C**), EAE mice in the remission phase (**D**–**F**) and minocycline-treated remitting EAE mice (**G**–**I**). (**J**) Histogram showing the microglial reactivity quantification in terms of area immunolabeled by CD68 in control mice, remitting EAE mice and remitting EAE mice treated with minocycline. Data are presented as the mean values of CD68 positive areas ± SEM. Please note that the intense microglial reaction observed in the remission phase of EAE is prevented by the treatment with minocycline. (**K**) Histogram showing the microglial activation in terms of optical density for CD68. Each value is the mean ± SEM. (**L**) Western blot analysis of CD68 in the three experimental groups (n = 3 for each group). (**M**) Analysis of the actin-normalized optical density of the bands showed a significant increase of CD68 expression in EAE mice when compared to controls and subsequent decrease of the expression after minocycline treatment (mean + SEM). The reported P-values are relative to within group comparisons (Tukey’s range test, ***P < 0.001, **P < 0.01, *P < 0.05).

It has been demonstrated that during the acute relapsing phase of experimental multiple sclerosis (EAE, clinical score above 3–4, see Methods) hippocampal microglial cells become activated^7^. We have now investigated whether the activation of microglial cells persists in the remission phase of the disease, after the resolution of motor deficits (Fig. 1). For the detection of activated microglia we performed CD68 immunostaining of our samples. To estimate the microglia reactivity in the CA1 hippocampal area, we detected both proliferation and morphological changes by quantifying the total surface covered by CD68. In control mice, only quiescent microglia was observed (Fig. 1A–C). Conversely, in the remission phase of EAE we observed an intense microglial reaction in terms of percentage of area stained with CD68 (P < 0.001, post hoc test) (Fig. 1D–F,J). The analysis of optical density confirmed the obtained results (P < 0.001, post hoc test) (Fig. 1K). These data suggest that activation of microglial cells persists in the EAE brain beyond the resolution of quantifiable neurological motor deficits in the utilized model.

In order to prevent the activation of microglial cells during the remission phase of EAE we investigated the effect of minocycline, an antibiotic with profound anti-inflammatory effects, being able to prevent microglial activation and to modulate T lymphocytes^8^.

The analysis of CD68 labelled area and optical density revealed no significant differences between control mice and remitting EAE mice treated with minocycline in terms of microglial reaction (P > 0.05) (Fig. 1G–I,J,K), suggesting that the treatment with minocycline, initiated at day 10 p.i., is able to prevent microglial activation in the utilized experimental model. Western blot analysis confirmed the upregulation of CD68 during the remission phase of EAE (P < 0.01, post hoc test). In particular, CD68 was found to be markedly increased in the hippocampus during EAE (Fig. 1L,M) and to be significantly decreased after the treatment with minocycline (P < 0.05, post hoc test).

### Impairment of synaptic LTP persists beyond the resolution of motor deficits in the hippocampal CA1 area of EAE mice

**Figure 2:**
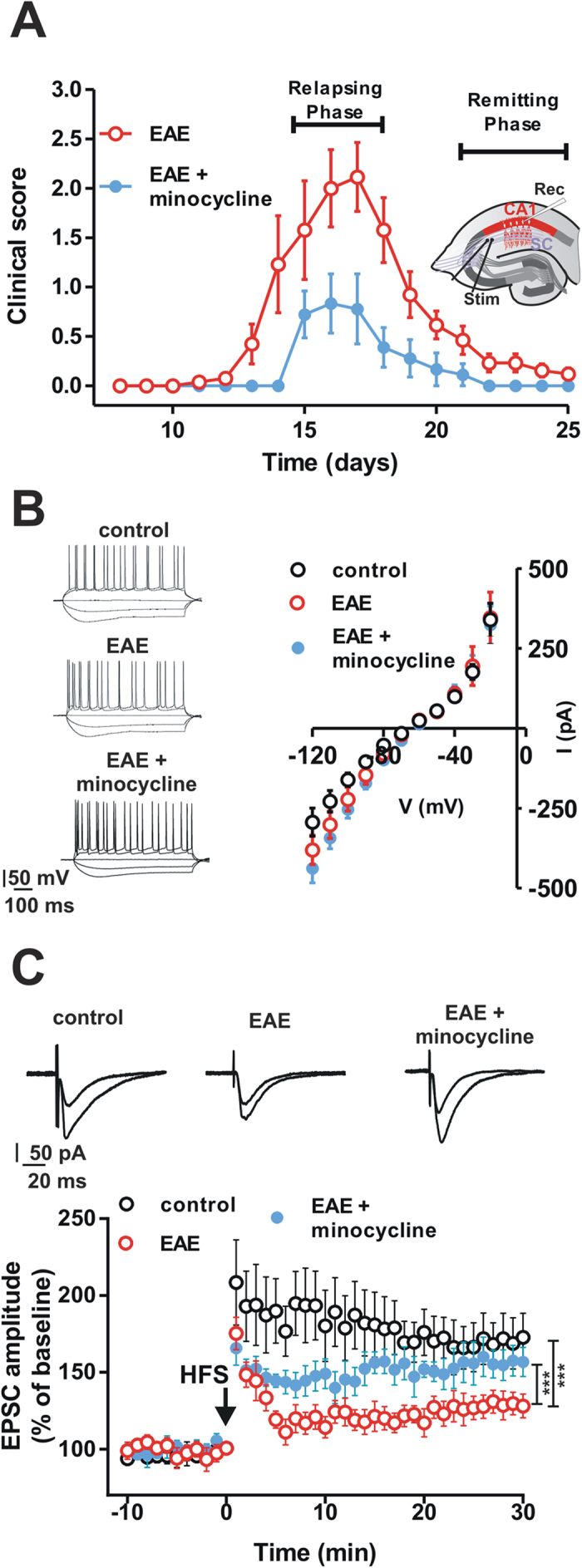
Hippocampal LTP is impaired in the remission phase of EAE and is restored by minocycline. (**A**) Graph showing the time-course of the mean neurological scores of EAE mice in control conditions (red symbols) and after treatment with minocycline (blue symbols). Note the reduction of the mean neurological scores during the relapsing phase of the disease in the minocycline-treated group. The inset shows the timing of electrophysiological recordings (remitting phase) and a scheme of a hippocampal slice showing the location of recording and stimulating electrodes. (**B**) Voltage traces (left) obtained from single CA1 hippocampal neurons patch-clamped in control condition, in the remission phase of EAE and in remitting EAE mice treated with minocycline. The graph on the right shows the I-V curve plots measured in CA1 hippocampal neurons from control, remitting EAE mice and remitting EAE mice treated with minocycline. (**C**) Graph showing the time-course of the EPSC amplitude recorded from CA1 hippocampal neurons in control mice (black symbols) compared with remitting EAE mice (red symbols) and remitting EAE mice treated with minocycline (blue symbols). Upper example EPSC traces before and after the HFS in the three experimental groups. Note the alteration of LTP in EAE mice and the restoring effect of minocycline. (***P < 0.001).

In order to investigate whether an alteration of the neuronal ability to express long-term changes of synaptic strength was present in the remission phase of EAE, we performed a single-cell patch clamp electrophysiological analysis of CA1 pyramidal neurons at this stage of the disease. CA1 pyramidal neurons were patch-clamped in slices from remitting EAE and control mice. No significant differences in the basal membrane properties were observed between neurons recorded in slices from remitting EAE (n = 5) and control mice (n = 5; P > 0.05) (Fig. 2B). For each experiment, after recording a stable evoked EPSC for 10 minutes, a high-frequency stimulation protocol was delivered to the slice in order to induce synaptic LTP. Hippocampal CA1 LTP recorded in neurons from remitting EAE mice (n = 7) was significantly reduced with respect to that observed in neurons of control mice (n = 6) (control 172.8 ± 15.68%; EAE 128 ± 7.7%; group F_(1,410)_ = 177.1, P < 0.001) (Fig. 2C). These data suggest that in the remission phase of EAE hippocampal CA1 synapses are not able to fully express long-term plasticity.

### CA1 LTP impairment is accompanied by cognitive deficits in remitting EAE mice

**Figure 3:**
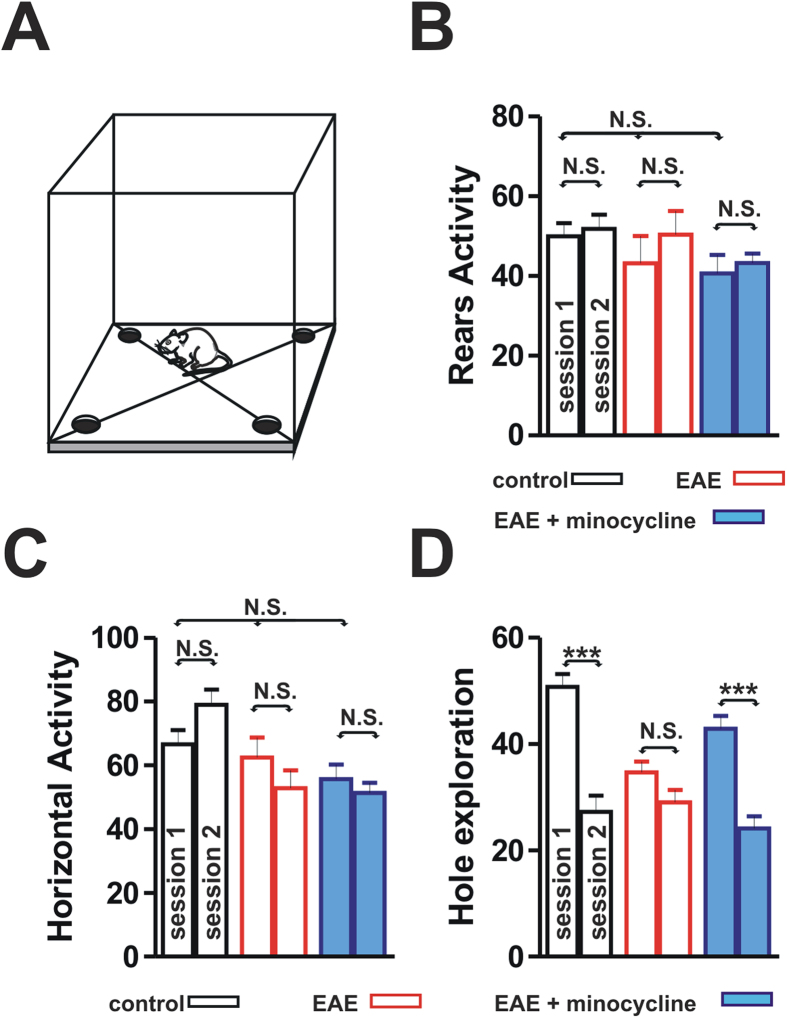
The performance of EAE mice in the hole-board learning test is impaired but restored by the minocycline treatment. (**A**) Scheme of a hole-board apparatus that includes four sectors and holes in the floor, for measuring horizontal motor activity, vertical motor activity (rearings) and nose pokes. (**B–D**) Histograms showing vertical motor activity as number of rears (**B**), horizontal locomotor activity as number of intertrial crossings (**C**) and hole exploration activity as number of head-dippings (**D**) during a 2-session holeboard test performed in control mice, remitting EAE mice and remitting EAE mice treated with minocycline. Note that vertical and horizontal activities (**B,C**) are not significantly different in the three experimental groups. (**D**) Control mice showed significantly less head-dippings when re-exposed to the hole-board compared with their initial performance. Conversely, EAE mice in the remission phase explored the holes to an equal degree after hole-board re-exposure, suggesting the presence of a learning/recognition deficit. The minocycline-treated remitting EAE mice showed a performance that was not different from that of control mice (***P < 0.001).

In order to explore whether the CA1 LTP impairment is associated with cognitive deficits during EAE, we measured the ability of both remitting EAE mice (n = 13) and control Biozzi ABH mice (n = 12) to recognize environmental spatial novelty by utilizing an open field hole-board test. This test has been demonstrated to involve the hippocampus^9^. In this test the number of head-dippings into holes is expression of object exploration. When habituation occurs, test re-exposure should lead to significantly fewer head-dippings^9^. No significant differences in horizontal locomotor activity and rears were observed between remitting EAE mice and controls (P > 0.05), suggesting that the overall motor activity was not altered in the remission phase of EAE (Fig. 3B,C). Specifically, horizontal locomotor activity and rears in the session 1 of the hole-board test were not significantly different between control mice, remitting EAE mice and minocycline-treated EAE mice (P > 0.05). Moreover, within each of the three experimental groups we did not find significant differences in horizontal locomotor activity and rears between the session 1 and the session 2 of the hole-board test (P > 0.05).

Conversely, significant differences were observed regarding holes exploration levels. In particular, control Biozzi mice showed significantly less head-dippings when re-exposed to the hole-board compared with their initial performance (session 2 vs session 1) while remitting EAE mice explored the holes to an equal degree after hole-board re-exposure (group X session, F_(2,30)_ = 13.25, P < 0.001) (Fig. 3D), suggesting that learning and recognition occurred in controls but not in EAE mice (group F_(1,12)_ = 58.67, P < 0.001). Remitting EAE mice explored less the holes already in the first day compared with controls (P < 0.001, post-hoc test) suggesting that EAE mice, in the context of sickness behaviour, probably also had difficulties in detecting the novel contextual change occurring during the hole-board test.

### NADPH oxidase mediates hippocampal synaptic plasticity deficits in the remission phase of EAE

We demonstrated that in the remission phase of EAE persistent microglial activation and cognitive/behavioural alterations are associated with an alteration of hippocampal synaptic plasticity. Thus, we turned attention to the mechanisms potentially underlying the observed deficit in neuronal plasticity.

It is well known that activated microglial cells can produce several factors that may damage neurons and synapses, such as pro-inflammatory cytokines and reactive oxygen species (ROS)^10^. NOX2, also known as GP91, is the catalytic subunit of the phagocyte nicotinamide adenine dinucleotide phosphate (NADPH) oxidase enzyme complex, that is highly expressed by activated microglia^11^. NADPH oxidase indeed transfer electrons across the plasma membrane from NADPH to molecular oxygen and may generate the free-radical superoxide and its downstream ROS^12^, playing a crucial role during the inflammatory process and chronic neurodegeneration^12^.

**Figure 4:**
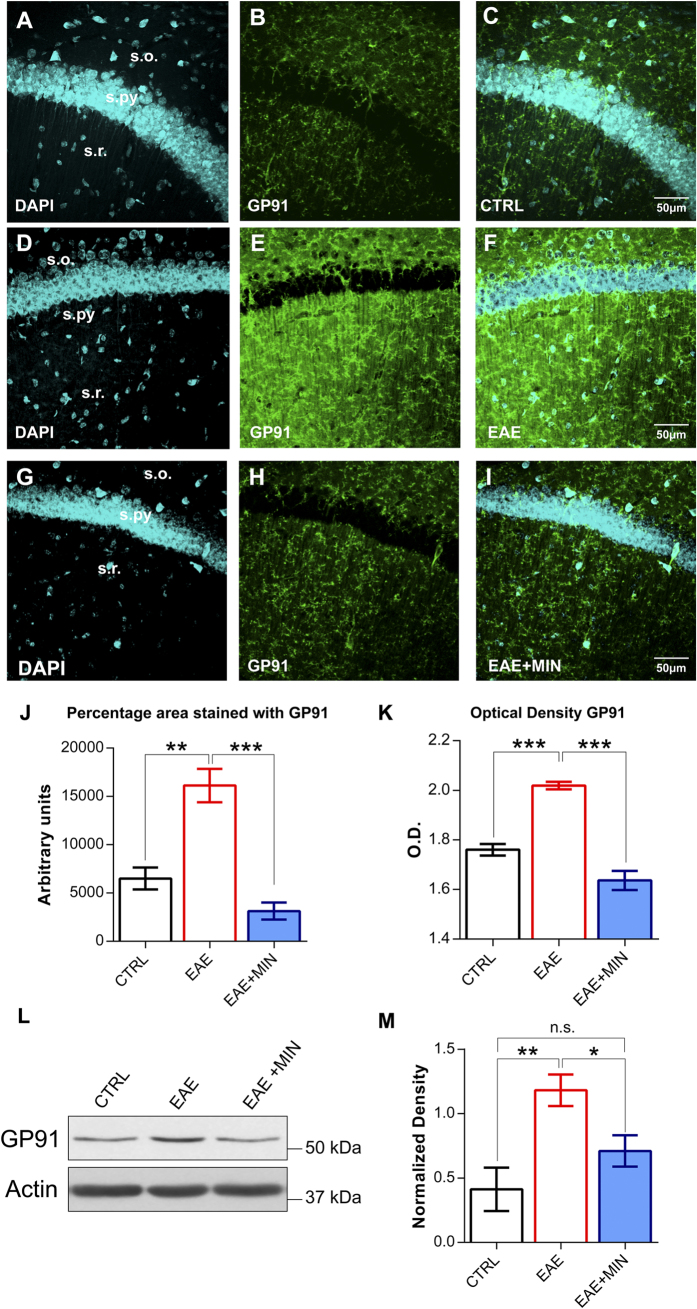
NADPH oxidase overexpression in the remission phase of EAE is prevented by minocycline treatment. (**A–I**) Confocal laser scanning microscopy (CLSM) images of double-label immunofluorescence for DAPI (visualized in blue) and GP91 (visualized in green-Cy2 fluorescence) in the CA1 hippocampal region from control mice (**A**–**C**), EAE mice in the remission phase (**D**–**F**) and minocycline-treated remitting EAE mice (**G**–**I**). (**J**) Histogram showing the quantification of GP91 in terms of percentage of area immunolabeled in control mice, remitting EAE mice and remitting EAE mice treated with minocycline. Data are presented as the mean values of GP91 positive areas ± SEM. Please note that NADPH oxidase expression is increased in the remission phase of EAE and the preventive effect of the treatment with minocycline. (**K**) Histogram showing the quantification of the optical density for GP91. Each value is the mean ± SEM. (**L**) GP91 expression analyzed with immunoblotting in the three experimental groups. On the right side (**M**) the histogram of actin-normalized optical density of GP91 in each group is depicted (mean + SEM, n = 3 for each group). The results confirmed the pattern of expression shown in immunohistochemistry. The reported P-values are relative to within group comparisons (Tukey’s range test, ***P < 0.001, **P < 0.01, *P < 0.05).

We have thus performed GP91 immunostaining, in order to investigate if the activation of microglial cells observed in the CA1 region of the hippocampus was accompanied by a parallel increase in NADPH oxidase expression. In the remission phase of EAE we observed a marked increase in GP91 expression with respect to control conditions (n = 4 for each group, P < 0.01, post hoc test) (Fig. 4A–F,J,K), suggesting that NADPH oxidase overexpression in the hippocampus accompanied the remission phase of EAE. Western blot analysis confirmed the upregulation of GP91 during the remission phase of EAE (Fig. 4L,M) (P < 0.01, post hoc test).

**Figure 5:**
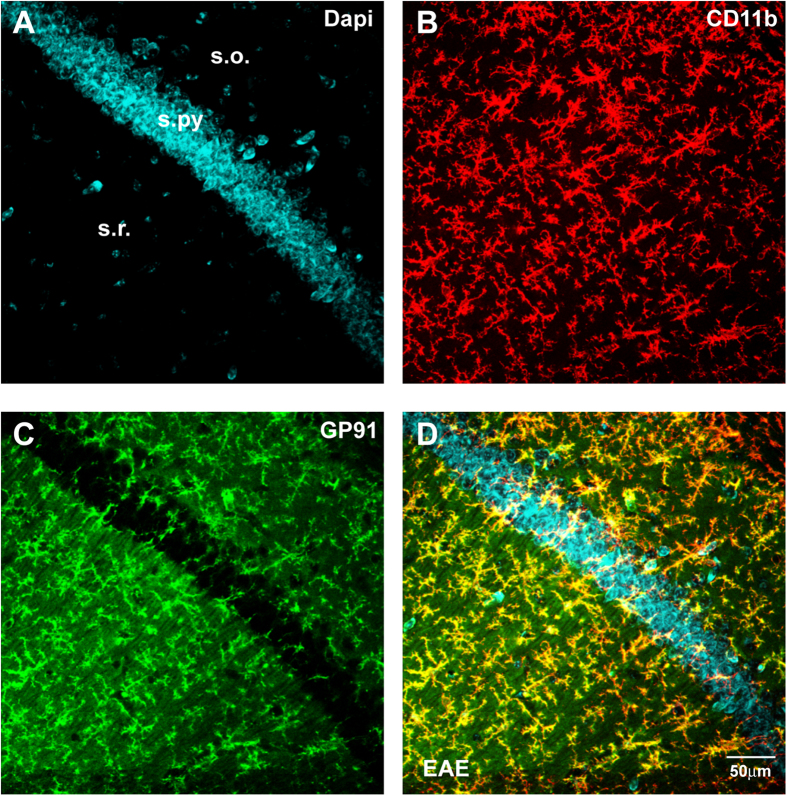
(**A**–**D**) Representative confocal laser scanning microscopy images of dual-label immunofluorescence for Cd11b and GP91 counterstained with DAPI in a CA1 sample from an EAE mouse in the remission phase. CD11b immunolabeling is revealed by red Cy3 immunofluorescence (panel **B**); GP91 is revealed by green Cy2 immunofluorescence (**C**). Colocalization is seen in yellow in the merged panel (**D**).

In order to confirm the cellular source of the expression of GP91 immunostaining, we performed a double immunofluorescence for CD11b and GP91. The immunofluorescence study showed that GP91 colocalized with the microglial cells immunolabeled for CD11b (Fig. 5).

Interestingly, in the experimental group treated with minocycline, the analysis of GP91 labelled area and optical density revealed no significant differences if compared with control mice in terms of NADPH oxidase expression (n = 5, P > 0.05) (Fig. 4G–I,J,K), suggesting that the treatment with minocycline is able to block the overexpression of NADPH oxidase that occurs in the remission phase of EAE. Quantification of GP91 with immunoblotting showed similar results to immunohistochemistry. GP91 was indeed found to be significantly increased during EAE (P < 0.01 post hoc test, Fig. 4L,M) and decreased in the experimental group treated with minocycline (P < 0.05, post hoc test). No significant differences were found between control mice and mice treated with minocycline (P > 0.05).

**Figure 6:**
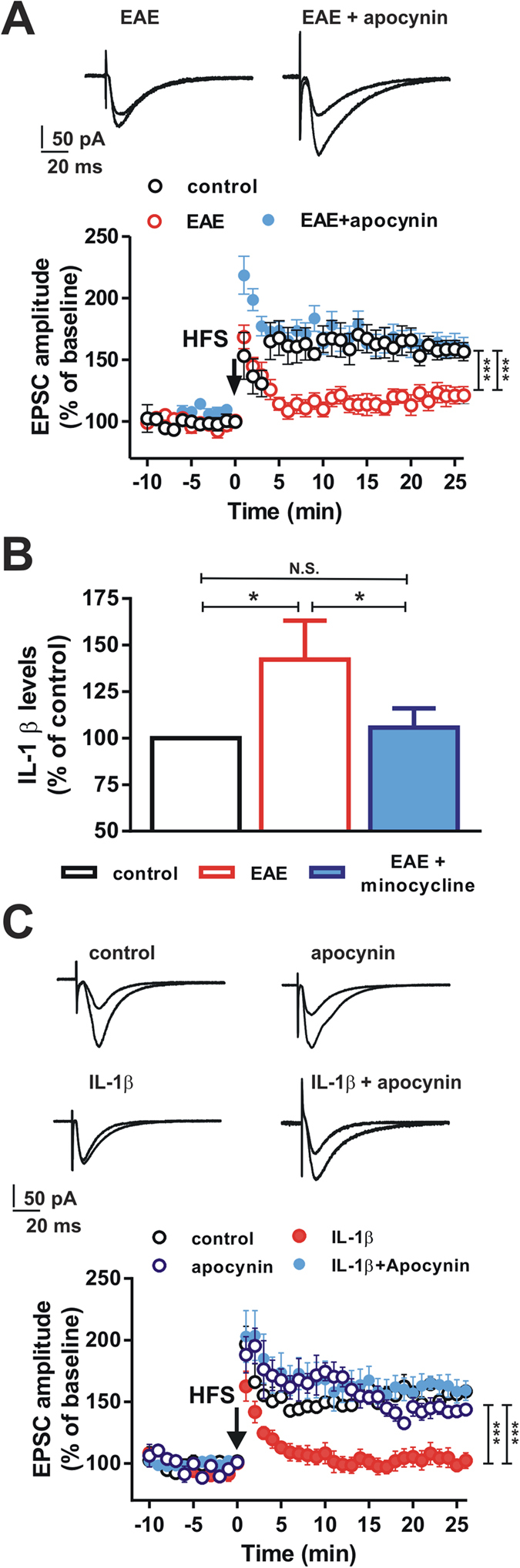
Inhibition of NADPH oxidase restores hippocampal plasticity in the remission phase of EAE and after IL-1β exposure. (**A**) Graph showing the time-course of the EPSC amplitude recorded from CA1 hippocampal neurons in control mice (open symbols), in remitting EAE mice (red symbols) and in remitting EAE mice in which the slices were incubated with apocynin (blue symbols). Example EPSC traces on the top are acquired before and after the HFS protocol in the two EAE groups. Note the reduced LTP in EAE mice and the restoration of the LTP produced by apocynin in EAE mice. (**B**) Histogram showing the IL-1β levels in controls, EAE mice and EAE mice treated with minocycline. (**C**) Time-course of the EPSC amplitude recorded from CA1 hippocampal neurons of control mice (open symbols), in the presence of IL-1β (red symbols), in the presence of apocynin (open blue symbols) or in the presence of IL-1β plus apocynin (solid blue symbols). Example EPSC traces on the top are acquired before and after the HFS protocol in the four experimental groups. Note the reduced LTP amplitude of neurons in the presence of IL-1β and the restoration of the LTP of neurons incubated with in the presence of IL-1β plus apocynin. (*P < 0.05, ***P < 0.001).

We thus investigated whether the inhibition of NADPH oxidase by apocynin (100 μM) was able to rescue synaptic plasticity during the remission phase of EAE. Interestingly, we found that hippocampal CA1 LTP, recorded in neurons of remitting EAE mice after slices exposure to apocynin (n = 6) was not significantly different from that recorded in neurons from control mice (n = 9) (control 158. 2 ± 7.95%; apocynin-exposed neurons 162.3 ± 9.04%; P > 0.05) (Fig. 6A), suggesting that the impairment of hippocampal synaptic plasticity observed in remitting EAE mice is mediated by NADPH oxidase.

### Increased levels of hippocampal interleukin-1β are associated with activation of microglia and hippocampal synaptic deficits during remitting EAE

As introduced above, activation of microglia is accompanied by release of pro-inflammatory molecules, such as interleukin**-**1β (IL-1β). Increased levels of IL-1β in CNS have been found during EAE^7^ and the cytokine is able, *per se*, to alter the expression of hippocampal synaptic plasticity^13^. Thus, we investigated if also the remitting phase of EAE was accompanied by a persistent increase in hippocampal levels of this pro-inflammatory molecule. We found that IL-1β levels were increased by ~1.5 fold in the hippocampus of remitting EAE mice, when compared to control animals (n = 6, P < 0.05, post-hoc test) (Fig. 6B). The analysis of the hippocampal levels of IL-1β was also performed in an experimental group treated with minocycline and showed no significant difference between minocycline-treated remitting EAE mice and control mice (P > 0.05; Fig. 6B), suggesting that the inhibition of microglial activation is also able to prevent the increase in hippocampal levels of IL-1β that is associated with the remission phase of EAE.

In order to explore the potential link between the hippocampal LTP deficit, increased IL-1β levels, and NADPH oxidase we investigated the effect of IL-1β on CA1 hippocampal plasticity. We found that exposure of hippocampal slices (n = 6) to IL-1β (60 pg/ml) mimicked the deficit of LTP observed in the remission phase of EAE (control 156.2 ± 4.026%; IL-1β-exposed neurons 98.45 ± 4.832%; group F_(1, 407)_ = 410.9, P < 0.001) (Fig. 6C). Interestingly, inhibition of NADPH oxidase, achieved by the co-application of apocynin, was able to reverse the block of LTP induction caused by IL-1β (n = 6) (IL-1β plus apocynin-exposed neurons 158.4 ± 9.396%; with respect to control P > 0.05) (Fig. 6C), suggesting that also the blocking effect of this pro-inflammatory cytokine on hippocampal plasticity is dependent on NADPH oxidase.

### Preventing microglia activation reverses hippocampal synaptic deficits in remitting EAE mice

Since microglial cells have the potential to influence synaptic function^14^, we investigated whether the prevention of microglia activation could result in the amelioration of synaptic deficits in remitting EAE mice. As described above, the treatment with minocycline prevented microglia activation in remitting EAE mice. Single cell patch clamp electrophysiological recordings in the CA1 area of the hippocampus have been thus performed to verify the ability of synapses to express LTP in remitting EAE mice treated with minocycline. Hippocampal CA1 LTP recorded in neurons from minocycline-treated EAE mice (n = 5) was not significantly reduced with respect to that recorded in neurons from controls (n = 6) (control 172.8 ± 15.68%; minocycline-treated EAE 156.7 ± 9.5%; P > 0.05) (Fig. 2C), suggesting that the inhibition of microglia activation prevented the synaptic deficits observed in remitting EAE mice.

**Figure 7:**
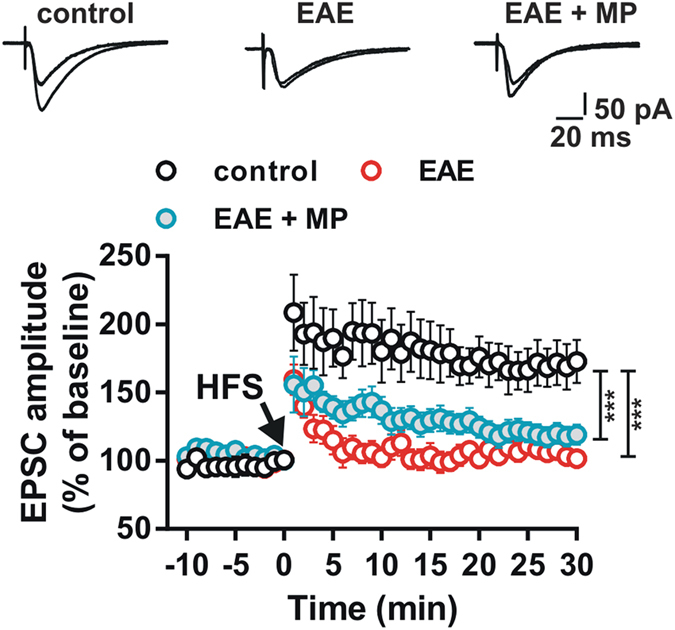
Relapse treatment with methylprednisolone does not prevent hippocampal plasticity deficits in the remission phase of EAE. Graph showing the time-course of the EPSC amplitude recorded from CA1 hippocampal neurons in control mice (open symbols), in remitting EAE mice (red symbols) and in remitting EAE mice treated with methylprednisolone (MP, blue symbols). Example upper EPSC traces are acquired before and after the HFS protocol in the three experimental groups. Note the reduced LTP in neurons of EAE mice and lack of restoration of LTP in mice treated with methylprednisolone. (***P < 0.001).

Currently, high dose methylprednisolone (MP) is the most utilized therapy for the treatment of multiple sclerosis relapses^15^. Thus, we investigated if the treatment of the EAE relapse with MP was able to prevent the synaptic hippocampal LTP deficit during the remission phase of the experimental disease. We found that hippocampal CA1 LTP recorded in neurons from EAE mice treated with MP (n = 14) was still reduced with respect to that observed in neurons of control mice (n = 6) (control 166.3 ± 15.91%; MP-treated EAE 121 ± 7.363%; group F_(1, 697)_ = 165.8, P < 0.001) (Fig. 7), suggesting that the acute treatment of the relapse with a course of MP is not able to prevent the synaptic CA1 hippocampal deficit.

### Prevention of microglia activation and restoration of CA1 LTP are associated with reversal of learning deficits in remitting EAE mice

Since the prevention of microglial activation was found to reverse the hippocampal LTP deficit, we also investigated if treatment with minocycline resulted in the amelioration of behavioural deficits in remitting EAE mice. During the hole-board test minocycline-treated EAE mice (n = 11) showed a performance that was not different from control mice. Minocycline-treated mice showed significantly less head-dippings when re-exposed to the hole-board compared with their initial performance (P < 0.001) (Fig. 3D), suggesting that the learning deficits associated with the remission phase of EAE were prevented by the treatment with minocycline.

## Discussion

The results obtained in the present study demonstrate that in the remission phase of experimental multiple sclerosis, CA1 hippocampal synapses fail to fully express long-term plasticity and that this synaptic dysfunction occurs in association with an impairment of cognitive performances. The synaptic and cognitive deficits were observed in the remission phase of the experimental disease, after the spontaneous resolution of EAE-associated motor deficits. This evidence is in line with the observation that neuropsychological dysfunction, and particularly memory deficits, persists in the remission phase in multiple sclerosis patients, after acute relapses^16^. Accordingly, cognitive deficits are evident in remitting multiple sclerosis patients, especially when brain MRI demonstrates underlying inflammatory disease activity^17^.

Interestingly, the alteration in synaptic plasticity was observed in the hippocampus, a key neuronal structure for cognitive functioning that is particularly vulnerable to the detrimental effects of neuro-inflammation. We found an impaired LTP in the CA1 area of the hippocampus, a brain region that is essential to encode spatial information^18^. Notably, structural MR studies have demonstrated the vulnerability of the hippocampus during the course of multiple sclerosis^19^, with the CA1 area being particularly involved^6^. Hippocampal glutamate levels have been found to correlate with visuo-spatial memory performances in multiple sclerosis patients^20^ and hippocampal plasticity is known to be negatively influenced by both inflammation and several pro-inflammatory immune molecules, such as interleukin-1β^3^,^4^,^7^,^13^,^21^.

Although the hippocampus seems to be particularly vulnerable during inflammatory processes it is worth to note that during EAE, abnormalities in synaptic plasticity have been described also in other brains structures, such as the cerebellum and the superior colliculus^4^. Thus, it is conceivable that the pathologic processes accompanying experimental MS diffusely alter synapses’ ability to express plastic changes, with subsequent functional alterations of different brain networks.

Cognitive impairment is a frequent concomitant of multiple sclerosis and it can be disabling. Multiple sclerosis–associated cognitive dysfunction appears early during the course of the disease^22^. Unfortunately, the precise mechanisms underlying cognitive impairment during the course of multiple sclerosis, and in particular their glial and neuronal substrates, are still under investigation. According to the present data, it is possible to hypothesize that an alteration of neuroplasticity may represent a contributing factor in multiple sclerosis-related cognitive impairment, as in other neurological diseases such as Alzheimer’s disease^23^ or Parkinson’s disease^24^. Accordingly, it is currently thought that, during multiple sclerosis, cognitive impairment is based on an alteration of the initial learning of information rather than on difficult retrieval from long-term storage^2^.

The obtained data suggest that, independently from the resolution of the motor deficits associated with the acute disease relapse, in the remission phase of EAE, synapses still fail to express LTP and cognitive/behavioural deficits may be unmasked by specific testing. It is worth to note that also in the very early phases of the experimental disease, i.e. before the appearance of motor deficits, spatial reference memory deficits have been detected^25^, suggesting that both the preclinical phase of EAE and its remission phase are associated with cognitive alterations.

In our model the observed deficits were found to depend on activation of microglial cells, since minocycline treatment, preventing microglial cells activation, reversed both the CA1 LTP deficit and the behavioural test alteration. Interestingly, although the treatment with minocycline ameliorated the clinical score of EAE mice, it did not completely prevent the development of the experimental disease, probably because the drug, at the utilized doses, only partially modulates the immunologic mechanisms involved in EAE pathogenesis.

In physiological conditions microglial cells dynamically interact with synaptic elements, with important effects in regulating synaptic connectivity^26^, synaptic pruning^27^, and learning-dependent synapse formation^28^. However, after activating stimuli, microglial cells have the potential to damage surrounding neurons and to mediate deficits in hippocampal synaptic plasticity^29^,^30^,^31^. Importantly, during EAE, the activation of microglia is found in association with synaptic alterations, atrophy of the pyramidal and dendritic layers of the hippocampal CA1 region, and impairments in AMPA and NMDA excitatory synaptic transmission^32^.

It is possible to hypothesize that the immune attack that characterizes the acute phase of EAE leads to an activation of microglial cells that persists beyond the resolution of motor deficits, releasing inflammatory mediators and altering synapses ability to express long-lasting memories. In this *scenario*, strategies aimed at preventing microglial activation may represent an important therapeutic tool.

We also found that, during the remission phase of experimental multiple sclerosis, microglial cells activation is accompanied by both an increase in hippocampal levels of the pro-inflammatory cytokine interleukin-1β and by the over-expression of the enzyme NADPH oxidase, that is able to generate ROS and to participate to neurodegenerative processes^12^. This evidence is particularly interesting because interleukin-1β was able, *per se*, to mimic the CA1 hippocampal LTP deficit observed during EAE and because it has been shown that ROS generation exceeding cellular antioxidant capacity is able to impair the induction of CA1 hippocampal LTP^33^. Interestingly, preventing the activation of microglial cells with minocycline normalized the expression levels of both interleukin-1β and NADPH oxidase and restored hippocampal plasticity. This latter evidence suggests that in the remission phase of EAE, the activation of microglia is associated with profound modifications of the brain microenvironment and in particular with an increase of potentially neurotoxic molecules.

In order to investigate the specific mechanism mediating the loss of synaptic plasticity during EAE, we tested the effect of an inhibitor of NADPH oxidase, apocynin, and found that it was able to restore the LTP deficit observed during the remission phase of experimental multiple sclerosis. This evidence points to the fact that hippocampal synaptic potentiation is altered during experimental multiple sclerosis by a mechanism involving the overexpression of ROS-producing NADPH oxidase by microglial cells.

Expression of NADPH oxidase has been recently analysed in multiple sclerosis lesions, in order to identify possible sources for ROS production in relation to demyelination and neurodegeneration^34^. Interestingly, various NADPH oxidase subunits and in particular GP91 and p22 were constitutively expressed in microglia and were up-regulated in the initial multiple sclerosis lesion, suggesting that oxidative burst through ROS production by NADPH oxidases is a key event during multiple sclerosis^34^. It is worth to note that the GP91 requires, for functional activation, the association with p47phox as a regulatory subunit^34^. Future studies should thus investigate in detail the role played by the different components of NADPH oxidase during EAE and multiple sclerosis.

Interestingly, apocynin was also able to prevent the impairment of LTP induced by interleukin-1β suggesting that the well-known blocking effect of this cytokine on synaptic plasticity is mediated by NADPH oxidase. Since it has been previously shown that microglial NADPH oxidase can be stimulated by interleukin-1β to produce hydrogen peroxide^35^, it is possible to hypothesize that during experimental multiple sclerosis, persistent inflammation and microglial activation may trigger an increase of interleukin-1β levels, NADPH oxidase over-expression and subsequent loss of neuroplasticity.

It is worth to note that, in our experimental model, the treatment of the acute EAE relapse with methylprednisolone, a therapeutic scheme usually utilized in the treatment of multiple sclerosis reactivation in daily clinical practice, did not prevent the development of hippocampal synaptic plasticity deficits. This latter evidence suggests that only a continuous treatment able to prevent the pathogenic cascade triggered by microglial activation can reverse the synaptic and memory deficits accompanying the remission phase of the disease. Accordingly, in other disease models, it has been demonstrate that drugs able to modulate the production of pro-inflammatory cytokines, are able to counteract alteration of synaptic plasticity by attenuating microglial activation^36^.

In conclusion, the present study demonstrates that even after the resolution of visible motor deficits, the EAE brain presents diffuse signs of persistent neuroinflammation, such as microglial activation, increased levels of pro-inflammatory cytokines and over-expression of neurotoxic ROS-producing enzymes. These elements are accompanied by synaptic deficits that appear to be mediated by NADPH oxidase and that are associated with cognitive/behavioural deficit. All together, these detrimental processes can be prevented by the inhibition of microglial activation, suggesting that the interaction between the immune and nervous system during multiple sclerosis deserves further studies, in order to define the precise molecular pathways to be targeted in order to restore long-term plasticity at CNS synapses during neuroinflammation.

## Methods

All procedures involving animals were performed according to the guidelines of University of Perugia Ethical Committee and the European Communities Council Directive 86/609/EEC.

### Induction of chronic-relapsing EAE

Chronic-relapsing EAE was chosen as experimental model of multiple sclerosis, since it predictably follows a clinical course reminiscent of relapse–remitting disease followed by disability accumulation^37^. For the induction of EAE, six to eight week-old female Biozzi ABH mice (Harlan UK Ltd., Bicester UK) were injected with 1 mg of syngenic spinal cord homogenate (SCH) in Freund’s adjuvant, as previously described^7^,^38^. Mice were injected with 1 mg of SCH emulsified in Freund’s incomplete adjuvant, supplemented with 100 μg of mycobacteria (*Mycobacterium tuberculosis* H37Ra and *M. butyricum* [8:1]) on day 0 and again on day 7. Animals were monitored and weighed daily, from day 10 post-inoculation (p.i.) onwards, to assess the development of relapsing-remitting paralysis. Clinical signs were scored as follows: 0 = normal; 1 = fully flaccid tail; 2 = impaired righting reflex; 3 = hindlimb paresis; 4 = complete hindlimb paresis; 5 = moribund/death^38^. The initial (acute) phase of EAE was anticipated to occur around 15–18 days p.i.^38^. In the EAE model, after this initial acute phase in which mice have variable clinical signs, the episode spontaneously resolves. In this remission phase of the disease (clinical score < 0.5) animals were selected for electrophysiological, behavioural and histological experiments and compared with age-matched control Biozzi ABH mice (Fig. 2A). In a subgroup of mice, minocycline hydrochloride was dissolved in PBS and administered daily by intraperitoneal (i.p.) injection. This treatment was initiated on day 10 post-immunization. EAE mice treated with minocycline received 50 mg/kg twice a day for the first 2 days, 50 mg/kg once a day for the next 5 days and 25 mg/kg once a day for the subsequent days. After having reached the remission phase of the disease animals were sacrificed. In the subgroup of mice treated with methylprednisolone (MP) [Solu-medrol^®^, Pfizer], beginning on the day of appearance of neurological signs, EAE mice were treated daily with i.p. injections of MP (50 mg/kg) for three days, until they were sacrificed, after having reached the remission phase of the disease. The mean neurological score of EAE mice on the day in which the treatment with MP was initiated was 2.6 ± 0.42. At the end of the treatment with MP the mean neurological score of EAE mice was 0.4 ± 0.15. Control EAE mice were injected daily with saline.

### Drugs

Freund’s incomplete adjuvant was purchased from Sigma-Aldrich (Milan, Italy); desiccated mycobacteria, *M. tuberculosis* H37Ra and *M. butyricum*, were from Difco laboratories (Detroit, U.S.A.); minocycline hydrochloride and apocynin were from Tocris Bioscience (Bristol, U.K.); IL-1β (60 pg/ml) was from R&D Systems (Minneapolis, U.S.A.).

### Immunohistochemistry

*Tissue processing.* Animals of the three experimental groups (control Biozzi mice, remitting EAE mice, and remitting EAE treated with minocycline) were transcardially perfused under deep anaesthesia with chloral hydrate with 60 mL of saline solution containing 0.05 mL heparin, followed by 200 mL of 4% paraformaldehyde in saline solution. Brains were removed and post-fixed overnight at +4 °C, cryoprotected in 20% sucrose and 10% glycerol in 0.1M phosphate buffer (PB) with sodium azide 0.02% for 48 hours at 4 °C. Subsequently, they were sectioned frozen on a sliding microtome at 40 μm thickness. To test the colocalization of GP91 with the microglial cell, a double immunofluorescence with mouse-anti CD11b (Immunological Science, Rome, Italy) and rabbit-anti GP91 was utilized. Subsequently, for the immunohistochemical detection of microglia activation, a double label immunofluorescence with rat anti-mouse-CD68 and GP91 antibody was employed. Briefly, sections were incubated with rat anti-mouse CD68 or rabbit anti-GP91 (Immunological Science, Rome Italy) at a 1:200 concentration in a 0.1M PB solution containing 0.3% Triton X and 0.02% sodium azide for 72 hours at + 4 °C. Sections were then rinsed three times for 15 minutes at room temperature and subsequently incubated with donkey anti-rat Cy3-conjugated secondary antibody (Jackson Immunoresearch, West Grove, PA, USA) or donkey anti-rabbit Alexa 488 (Immunological Science, Rome Italy) for 2 hours at room temperature. Sections were then rinsed two times and then DAPI counterstained. Images were acquired by using a Confocal laser scanning microscope (CLSM, Zeiss LSM 700), under non-saturating exposure conditions and using the same acquisition settings for all samples. Gain and laser power, were selected at levels that allowed optimal visualization of the fluorophore used as secondary antibody and standardized using sections from control mice. Each image was saved at a resolution of 1024 × 1024 pixels. These settings were then applied as standards for all subsequent images. Z-stacks images (40× objective) of CA1 coronal sections were collected using the computer-controlled microstepper stage of the confocal microscope. Stacks of images were combined into a single two-dimensional (2D) projection image, exported in TIF file format using NIH ImageJ software and used to quantify the area and the optical density (O.D.) of immunohistochemically positive tissue for CD68 or GP91. The area of immunolabeling was calculated in the three separated fields (dorsolateral, central and medial, 1 mm in diameter) on the hemispheres of three rostrocaudally spaced sections obtained from 4 mice of each experimental group. Before quantifying the OD in each image, we measured a mean grey value of the background and then performed an optical density calibration by means of a calibrated optical density step tablet. The resulting average OD in each section was taken as a measure for microglial activation.

The data obtained by image J analysis were compared by means of one- way ANOVA including the group (CTRL, EAE, EAE + minocycline) as main factor. Post hoc pair comparisons were carried out where necessary using Tukey’s range test. Values of P < 0.05 were considered to be statistically significant.

### Western blot

Hippocampi from the three experimental groups (n = 3 for each group, 20–40 mg wet weight) were lysed with 0.2 mL of RIPA buffer with the addition of 0.1% SDS. After manual homogenization with a potter on ice, the samples were tip-sonicated (3 times × 15 seconds) and left on ice for 15 minutes. Lysates were centrifuged for 10 minutes at 4 °C (14.000 × g) and the supernatants were collected. Protein concentration was measured with the Bradford assay. Fifty μg of proteins were separated on a 10% polyacrylamide gel for GP91 and 8% for CD68. The latter was run in non reducing conditions (no boiling, no reducing agents). Proteins were transferred on nitrocellulose membrane for 2 hours, which were probed overnight with anti-GP91 (Immunological Sciences, Rome, Italy) and anti-CD68 (clone FA-11, AbD Serotec, Puchheim, Germany) antibodies. Detection was carried out using the appropriate secondary antibody conjugated to horseradish peroxidase. Membranes were developed with ECL reagents. Actin and total Ponceau staining of each lane were used as loading controls and normalization factor for band optical density. ANOVA with Tukey’s range test for within groups comparisons were used for statistical analysis, P < 0.05 was deemed as significant.

### Electrophysiology

For hippocampal recordings mice were anesthetized with 2-bromo-2-chloro-1,1,1-trifluoroethane before decapitation. The brain was dissected and immersed in ice-cold artificial cerebrospinal fluid (ACSF) containing (in mM): 126 NaCl, 2.5 KCl, 1.2 MgCl_2_, 1.2 NaH_2_PO_4_, 2.4 CaCl_2_, 10 glucose, and 25 NaHCO_3_, continuously bubbled with 95% O_2_ and 5% CO_2_, pH 7.4. Transverse hippocampal (250 μm thick) slices were cut by using a vibratome and were allowed to recover in oxygenated ACSF at 30 °C for 30 minutes, and then at room temperature for another 1–2 hours before experimental recordings. Each slice was then transferred into the recording chamber and submerged in ACSF at a constant rate of 2.9–3 mL/min at a temperature of 29 °C. Under visual control, a stimulating electrode was inserted into the Schaffer collateral fibres, and a recording electrode into the CA1 region of the hippocampal slice. Whole-cell patch clamp recordings (holding potential, −60 mV) were performed with borosilicate glass pipettes. Neurons of the CA1 region were visualized using differential interference contrast (Nomarski) and infrared microscopy (Olympus). Excitatory postsynaptic currents (EPSCs) of half-maximal amplitude were evoked every 10 seconds; LTP was induced by a high-frequency stimulation protocol consisting of three trains at 100 Hz (1 second duration and 20 seconds interval). Two-way ANOVA was used for statistical analysis.

The significance level was established at P < 0.05. All experimental protocol was approved by the Bioethical Committee of the University of Perugia.

### Behavioural experiments

*Hole-board test.* The protocol used for the hole-board test was a modified version of the one described by Kemp *et al.* 2008^39^,^40^. The recording chamber measured 28 × 28 × 60 cm. The floor was made of an opaque grey PVC and the walls of transparent PVC panels. The top was closed with a transparent PVC panel provided of several holes for air circulation. In this apparatus a camera installed on the top of the chamber recorded the sessions. To enable proper acclimatization of animals, the home cages were transferred to the experiment room one hour before the experimental session. In the first day (habituation), habituation to the recording chamber was enabled, allowing the animals to explore the context for a 10 minutes session. After 24 hours, a hole-board (28 × 28 × 2.5 cm) was inserted into the floor of the recording chamber. Each corner of the hole-board contained a hole, 2 cm in diameter and 2.5 cm deep (Fig. 3A). Animals were allowed to explore the hole-board for 10 minutes (session 1), and the number of hole nose-pokes were counted. After 24 hour, the same experimental context was maintained, animals were allowed to explore for other 10 minutes (session 2) and the nose-pokes were recorded. To ensure that no odour cues interfered during the test, box and hole-boards were cleaned with alcohol (70%) and water solution after each trial. The locomotor activity was evaluated by an observer blind to the experimental group. The hole-board arena was divided into four equal sized squares and locomotion was measured as the number of crossings from one square to another. A blind observer also counted the number of rears, a parameter of vertical motor activity that reflects exploratory activity of the hole-board environment. Two-way RM-ANOVA was used to analyse statistical differences between groups, and Tukey’s range test for post-hoc comparisons.

### Measurement of interleukin-1β (IL-1β) levels in the hippocampus

Hippocampal slices were obtained from the three groups (control Biozzi mice, remitting EAE mice, and remitting EAE treated with minocycline, n = 6 for each group) as reported above. Four slices were pooled from each animal (total wet weight ~50 mg). Tissue slices were lysed in 0.3 mL of lysis buffer (Life Technologies, Carlsbad, CA, USA, product code FNN0021), first with a manual potter on ice, then tip-sonicated (3 times × 15 seconds) and left on ice for 15 minutes. Samples were centrifuged at 14,000 × *g* for 10 minutes at 4 °C and the supernatant collected and stored at −80 °C pending analyses. Total protein content was determined according to Bradford. IL-1β levels in the samples were measured using the xMAP™ technique^41^, with a kit specific for mouse IL-1β (Life Technologies). All measurements were performed at least in duplicate, following the instructions of the manufacturer. Solubilized samples (100 μL) were dispensed undiluted on the microtiter plate and incubated for 30 minutes at room temperature with beads conjugated with the specific monoclonal antibody for IL-1β. Biotinylated secondary antibody was then added and incubated with the beads at room temperature for 30 minutes. Streptavidin-conjugated phycoerytrin was added to all wells and incubated for 10 minutes at room temperature. After plate washing, samples were quantified using a Luminex 200 instrument (BIORAD Hercules CA, USA). Standard curves were built with calibrators included in the kit and diluted in lysis buffer. The same lysis buffer used for sample solubilisation was used as blank. Median fluorescence of the samples was interpolated using a five parameter logistic fit method embedded in the software Bioplex Manager v. 5.0 (BIORAD Hercules CA, USA). Data were normalized against the total protein content of each sample. ANOVA with Tukey’s range test for within groups comparisons were used for statistical analysis, P < 0.05 was deemed as significant.

## Additional Information

**How to cite this article**: Di Filippo, M. *et al.* Persistent activation of microglia and NADPH oxidase drive hippocampal dysfunction in experimental multiple sclerosis. *Sci. Rep.* **6**, 20926; doi: 10.1038/srep20926 (2016).
