# Erratum: Persistent activation of microglia and NADPH oxidase drive hippocampal dysfunction in experimental multiple sclerosis

**DOI:** 10.1038/srep23855

**Published:** 2016-04-13

**Authors:** Massimiliano Di Filippo, Antonio de Iure, Carmela Giampà, Davide Chiasserini, Alessandro Tozzi, Pier Luigi Orvietani, Veronica Ghiglieri, Michela Tantucci, Valentina Durante, Ana Quiroga-Varela, Andrea Mancini, Cinzia Costa, Paola Sarchielli, Francesca Romana Fusco, Paolo Calabresi

Scientific Reports 6: Article number: 2092610.1038/srep20926; published online: 02182016; updated: 04132016

The original version of this Article contained an error in the title of the paper, where “Persistent activation of microglia and NADPH oxidase drive hippocampal dysfunction in experimental multiple sclerosis” was incorrectly given as “Persistent activation of microglia and NADPH drive hippocampal dysfunction in experimental multiple sclerosis”. This has now been corrected in the PDF and HTML versions of the Article.

